# Case Report of a Dental Implant with Conometric Abutment–Prosthetic Cap Connection: Advanced High-Resolution Imaging and Peri-Implant Connective Tissue Performance

**DOI:** 10.3390/clinpract14020043

**Published:** 2024-03-27

**Authors:** Nicole Riberti, Emira D’Amico, Tania Vanessa Pierfelice, Michele Furlani, Alessandra Giuliani, Adriano Piattelli, Giovanna Iezzi, Luca Comuzzi

**Affiliations:** 1 Neuroscience, Imaging and Clinical Sciences Department, University of Chieti-Pescara, 66100 Chieti, Italy; nicole.riberti@unich.it; 2 Medical, Oral and Biotechnological Sciences Department, University of Chieti-Pescara, 66100 Chieti, Italy; emira.damico@unich.it (E.D.); tania.pierfelice@unich.it (T.V.P.); gio.iezzi@unich.it (G.I.); 3 Odontostomatologic and Specialized Clinical Sciences Department, Polytechnic University of Marche, Via Brecce Bianche, 60131 Ancona, Italy; m.furlani@pm.univpm.it; 4 School of Dentistry, Saint Camillus International University for Health Sciences (Unicamillus), 00131 Rome, Italy; adriano.piattelli@unicamillus.org; 5 Independent Researcher, Via Raffaello 36/a, 31020 San Vendemiano (TV), Italy; luca.comuzzi@gmail.com

**Keywords:** conometric prosthetic connection, peri-implant soft tissues, case report, artificial intelligence, imaging methods

## Abstract

**Background**: In recent years, the use of conometric systems to connect dental implant abutments and prosthetic caps has been advocated because they seem to eliminate the side effects reported when using screw- and cement-connected prosthetic restorations. **Objectives**: The present case study is focused on conometric connection characterization and its performance in terms of the microarchitecture of peri-implant soft tissues by using a cross-linked approach based on optical microscopy and three-dimensional imaging. **Methods**: Two dental implants were characterized using micro-CT and another identical one was implanted into a patient; the latter was retrieved 45 days later due to changes in prosthetic needs. Afterward, the peri-implant soft tissues were investigated using synchrotron-based phase contrast imaging, histology, and polarized light microscopy. **Results**: Micro-CT analysis showed perfect adhesion between the abutment and prosthetic cap; histology and polarized light microscopy showed that connective tissue was richly present around the abutment retrieved from the patient. Moreover, the quantitative evaluation of connective tissues using synchrotron imaging, supported by artificial intelligence, revealed that this tissue was rich in mature collagen, with longitudinal and transverse collagen bundles intertwined. The number and connectivity of transverse bundles were consistently greater than those of the longitudinal bundles. **Conclusion**: It was found that the peri-implant soft tissue was already mature and well organized after only 45 days of implantation, supporting the hypothesis that conometric connections contribute to the significant stabilization of peri-implant soft tissues.

## 1. Introduction

Several papers have recently shown that peri-implant connective tissues’ three-dimensional (3D) organization plays a key role in the preservation of peri-implant bone tissues and the long-term survival and success of dental implants [1,2,3,4,5]. Animal models and human studies have described the structure and ultrastructure of peri-implant soft tissues using bi-dimensional imaging methods like histology, scanning electron microscopy, polarized light microscopy [6,7,8,9,10,11], and transmission electron microscopy [12]. However, only advanced 3D imaging through high-resolution tomography (micro-CT) has been able to give evidence and quantify the organization of connective tissue microarchitecture [13,14,15].

Until recent years, prosthetic restorations were connected to implant abutments mainly by using screws or cement; however, both methods have some contraindications [16,17,18,19]. In screw-retained prosthetic restorations, there can be loosening or fracture of the screws; moreover, a not-so-optimal esthetic result is often obtained due to the presence of an occlusal access hole [17,18,19]. This access hole can also produce a weak spot, with possible chipping and fracture of the porcelain [20,21]. In cement-retained restorations, biological complications can occur, e.g., inflammation of the peri-implant soft tissues with loss at the level of the peri-implant crestal bone due to excess cement [17,18,19].

In the last decade, a conometric system has been proposed, by which the prosthetic crowns are coupled to the abutment using a precise friction fit, without screws or cement [22,23,24,25,26,27,28]. This connection, eliminating the cement residues in the subgingival region, has been proven to reduce the risk of inflammation of the peri-implant tissues. Moreover, by not having to remove cement residues, it is possible to set the margins in more apical portions, improving the aesthetics of the restorations. It has also been proven that retentive force is adequate for fixed rehabilitation even after a high number of insertion–disengagement cycles [29].

Several studies report successful follow-up evaluations of conometric systems [29]; some of these studies are accompanied by histological investigations mainly referring to the study of the peri-implant bone. Consequently, for full acceptance and recognition of the conometric abutment–prosthetic cap connection, it is necessary to further study the peri-implant soft tissues which constitute the actual barrier to the migration of any bacteria and inflammatory cells towards the apical bone areas.

According to the present authors’ best knowledge, there are no cases in the literature showing definitive results on the 3D structure of peri-implant connective tissues around conometric prosthetic restorations with proven absence of macro- and micro-gaps between the abutment and the prosthetic cap. The aim of the present case study is to shed light, through the use of micro-CT, on possible 3D defects in conometric abutment–prosthetic cap connections and, via histology, polarized light microscopy, and synchrotron-based micro-CT, the organization of peri-implant connective tissues around a retrieved human dental implant with conometric connection used for restoration.

## 2. Materials and Methods

### 2.1. Samples

**Figure 1:**
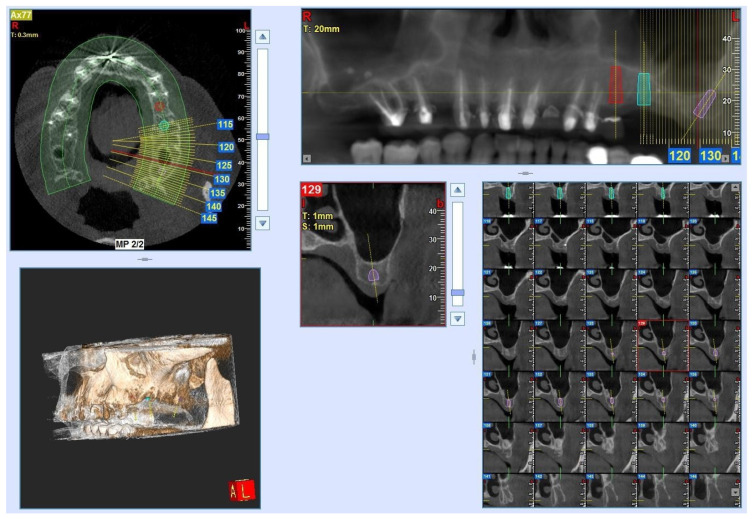
Cone–beam study (CBCT) (FOV 11 × 5 cm^2^). The resolution was 300 µm for planning implant rehabilitation.

**Figure 2:**
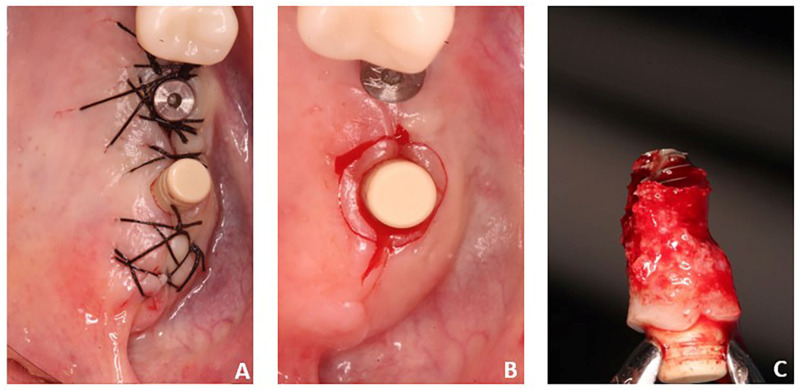
(**A**) Implants in sites 26 and 27 after insertion; (**B**) mucotomies around the implant after 45 days of healing; (**C**) implant and peri-implant soft tissues retrieved.

A 61-year-old female patient who had undergone implant rehabilitation received three (nr = 3 samples) dental titanium implants with conometric connection (AoN Implants, Grisignano di Zocco, Vicenza, Italy). The patient received a clear explanation of the study and signed an informed consent form. A cone–beam study was conducted before the surgery for the planning of implant rehabilitation (NewTom Giano with field of view (FOV) 11 × 5 cm^2^ and resolution 300 µm) (Figure 1). Implant rehabilitation was performed in the premolar–molar region of the left maxilla (sites 25-26-27). The three implants (AoN Implants, Grisignano di Zocco, Vicenza, Italy) were placed 1 mm subcrestally, and the conometric abutments, measuring 3 mm, were inserted in all sites and tightened at 25 N (Figure 2A,B). The drilling protocol involved the use of a 2 mm cylindrical dental drill and a dedicated 8 × 3.3 mm^2^ conical drill at 50 rpm without irrigation. The implant was inserted into soft bone (type IV) at 15–18 N. The retrieved implant was an AoN Is-Four implant, measuring 3.3 mm in width by 8 mm in length. It consisted of a titanium, conometric straight abutment (5 mm high) and a conometric cap made of PEEK. The PEEK cap was used over a metal one to offer a less bulky alternative during this temporary phase.

**Figure 3:**
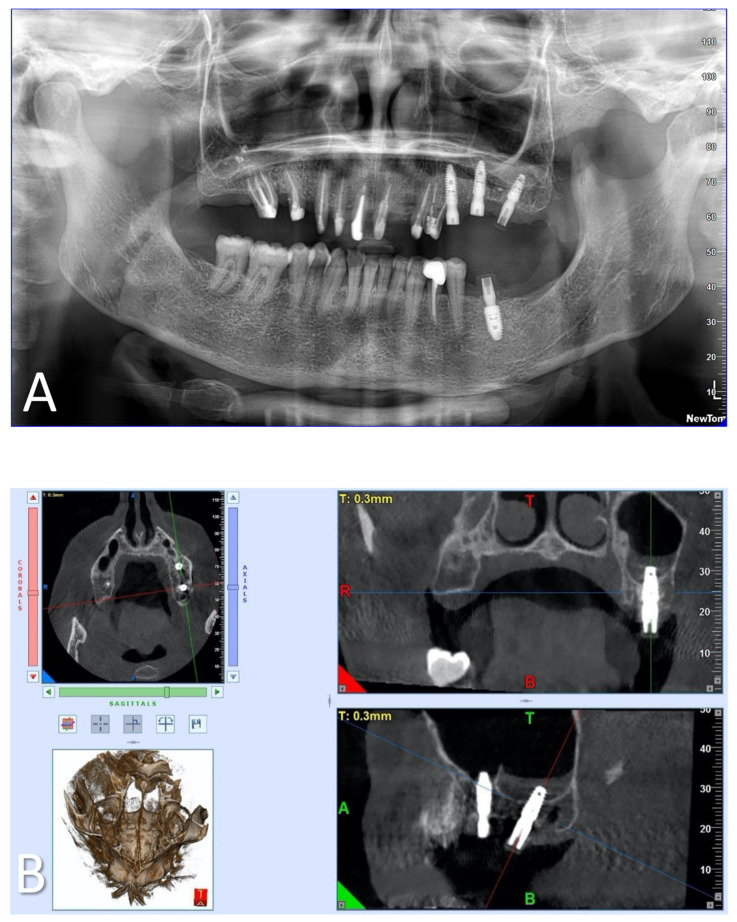
(**A**) OPT study after 45 days of healing and (**B**) cone–beam study after 45 days of healing: blue: axial planes; red: coronal planes; green: sagittal planes; T: top; B: bottom; R: right; L: left; A: anterior; P: posterior.

**Figure 4:**
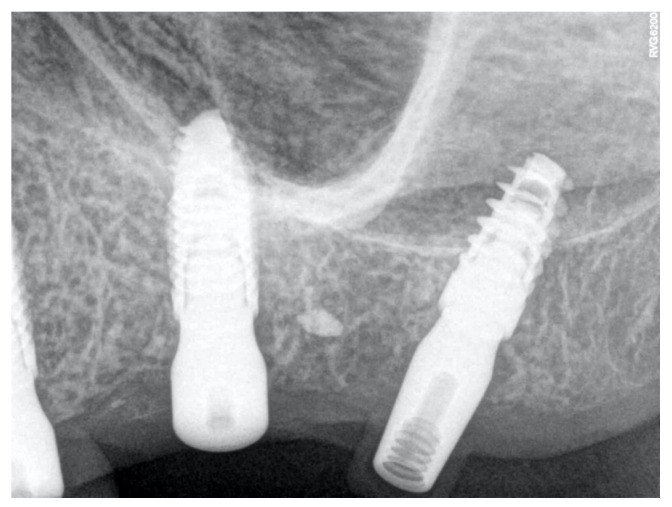
Radiography of the implant after 45 days of healing.

After suturing, wound healing remained undisturbed for 45 days. Therapy included clavulanic acid and amoxicillin at 1 g for 6 days, prednisone at 5 mg for 4 days, and paracetamol at 1 g if necessary. At the end of the operation, dexamethasone at 5 mg was administered to contain post-operative swelling. After implant rehabilitation, a radiographic investigation was conducted using orthopantomography (OPT) and cone–beam study (Figure 3). After a period of 45 days, one of these implants—site 27—together with the peri-implant tissues was retrieved. Indeed, it was positioned distally far, causing discomfort and interfering with her speech and tongue movement. Therefore, a change in prosthetic rehabilitation was necessary (Figure 2C and Figure 4). In detail, implant retrieval was performed via a parallel incision to the implant axis at 2 mm from the abutment in the mucosal tissue, using a magnetic mallet for the bone tissue. This achieved a cylinder core of tissue of 8–10 mm thickness in the axial direction. In addition, a gingivectomy was necessary near to where the implant was located.

The Ethics Committee of the University of Chieti-Pescara approved the use of this specimen for research purposes (CODE: BONEISTO, 15 September 2019). The implant used in this work was delivered in 2019 to the Dental School of the University of Chieti-Pescara, Italy, to be stored. The specimen was already fixed with 10% buffered formalin. Then, dehydration was carried out with increasing concentrations of alcohol. Finally, the sample was embedded in glycol–methacrylate resin (Technovit 7200 VLC; Kulzer, Wehrheim, Germany) before being stored in the archives. The specimen was processed after being retrieved from the archives in 2023, in accordance with Ref [13]. The synchrotron and histological analyses were performed on the same sample. In brief, it was sectioned into two parts along its longitudinal axis. The first part was used to examine the implant’s peri-implant tissues using synchrotron micro-CT after abutment removal; after micro-CT, this part was further sectioned along the transversal axis for histological analysis. The second portion of the sample was used to obtain histological longitudinal sections of the peri-implant tissues. This sample was already included in a prior study to validate an artificial intelligence (AI) module, not for diagnostic purposes [15]. The CARE Checklist is reported as Table S1 in Supplementary Materials.

Two other (nr = 2 samples) identical titanium implants with conometric connection were received by AoN Implants (Grisignano di Zocco, Vicenza, Italy) for implant characterization using laboratory-based micro-CT. The implants had the abutment fixed in a plexiglass support and the conometric cap was pre-assembled.

### 2.2. Laboratory-Based Micro-CT Investigation of the Implant

The X-ray micro-CT analysis was performed on nr.2 commercial implants as such using a Bruker Skyscan 1174 tomographic system (Bruker, Kontich, Belgium). In order to carry out the volumetric analysis, the two systems, taken from two different productions to verify their reproducibility, were pre-assembled with the abutment in the plexiglass support and already connected with the conometric cap. The analysis was performed to detect eventual defects (gaps) in the conometric abutment–prosthetic cap connection.

The micro-CT system was set with a power voltage of 50 kV and a beam current of 800 µA. Parameter settings: pixel size = 6.5 µm; 1.5 mm Al filter on the source X-ray beam; and exposure time = 17.5 s. The samples were scanned over 180° using a 0.3° scan step. After the tomographic scan, the reconstruction phase was carried out with the Bruker NRecon software (v. 1.7.3), setting the smoothing algorithm (3.0), ring artifact reduction (5.0), and beam hardening correction (10%).

### 2.3. Synchrotron Light-Based Phase-Contrast Micro-CT of Peri-Implant Connective Tissue

The sample retrieved from the 61-year-old female patient 45 days after implantation surgery was investigated via synchrotron-based micro-CT.

The micro-CT experiment was conducted at the SYRMEP beamline of the ELETTRA Synchrotron Radiation Facility (Basovizza (TS), Italy). The parameters were set as follows: 1800 projections, each with an exposure time of 0.2 s; a sample–detector distance of 100 mm; a total angular range of 180°; peak energy at 17 keV; and a pixel size of 890 nm. The coherence characteristics of the synchrotron’s light allowed for use of a propagation-based phase contrast setting at high resolution. The index of refraction *n* = 1 − *δ + i β* was reconstructed; in this formula, the phase shift term (*δ*) is proportional to the tissue electron density, whereas the complex part *β* is proportional to the tissue density. The *δ/β* ratio was established at 100, after several tests. To recover the various phases (vessels, collagen, etc.), the Paganin method was utilized [30]. Data processing was carried out using ORS Dragonfly software (Version 2022.1; Object Research Systems, Montreal, QC, Canada) [31]: indeed, its anisotropy and deep learning tools are capable of employing different algorithms to analyze and segment images with artificial intelligence. The collagen bundles were subsequently analyzed morphometrically with the BoneJ plugin [32] within the Fiji platform [33]. These software tools computed several structural indices: the specific volume of collagen (expressed as a percentage), i.e., the amount of collagenous tissue per unit volume; the density of connectivity (expressed as pixel^−3^) that quantifies how many collagen fibers are connected to each other, presenting as a connectivity index per unit volume; and the degree of anisotropy (DA), which provides information on the direction of the collagen bundles (the more anisotropic the object, the greater the DA value).

Four volumes of 600 × 300 × 300 pixels^3^ (approximately 40 × 10^−3^ mm^3^) were chosen close to the implant interface in order to generate a dataset that was as uniform as feasible and did not induce internal distortions. The artificial intelligence module of the ORS Dragonfly 2022.1 software was used to manually segment slices, as it contains all of the necessary tools to best isolate fibers (transverse and longitudinal) from the background. As described elsewhere [15], a network that employed a semantic segmentation of three classes was trained; this deep learning method allowed us to label each pixel based on the morphometric properties of the image. Therefore, if some objects in the foreground had a different orientation or shape, they were categorized into two distinct subgroups with two distinct labels. The neural network U-Net, devised by Olaf Ronneberger et al. [34] and specifically designed for biomedical image segmentation, was used in the semantic segmentation method.

### 2.4. Histological Investigation

For the histological investigation, the specimen was sectioned, along its longitudinal and transversal axis, with a high-precision diamond disk at about 150 µm and ground down to about 30 µm with a specially designed grinding machine, Precise 1 Automated System. The obtained slice was then stained with acid fuchsin and toluidine blue. Histological analysis was carried out using a light microscope (Laborlux S, Leitz, Wetzlar, Germany) connected to a high-resolution video camera (3CCD, JVCKY-F55B, JVC, Yokohama, Japan) and interfaced with a monitor and PC (Intel Pentium III 1200 MMX, Intel, Santa Clara, CA, USA). This optical system was associated with a digitizing pad (Matrix Vision GmbH, Oppenweiler, Germany) and a histomorphometry software package with image-capturing capabilities (Image-Pro Plus 4.5, Media Cybernetics Inc., Immagini & Computer Snc, Milano, Italy). The histological findings were reviewed by a single, highly qualified expert (G.I.), who was not engaged in the surgical procedure.

### 2.5. Polarized Light Microscopy

Using polarized light microscopy, the transverse orientation of collagen bundles was observed as a result of birefringence. The collagen fibers were viewed using an Axiolab light microscope (Laborlux S, Leitz, Wetzlar, Germany) equipped with two linear polarizers, two quarter wave plates, and circularly polarized transmitted light. As a result of a change in the refraction of existing light, the collagen fibers (bright fibers) were precisely aligned perpendicular to the direction of light propagation (parallel to the section plane). In contrast, the different-colored collagen fibers were aligned along the axis of light propagation (perpendicular to the section plane) and no refraction occurred.

## 3. Results

### 3.1. Laboratory-Based Micro-CT

**Figure 5:**
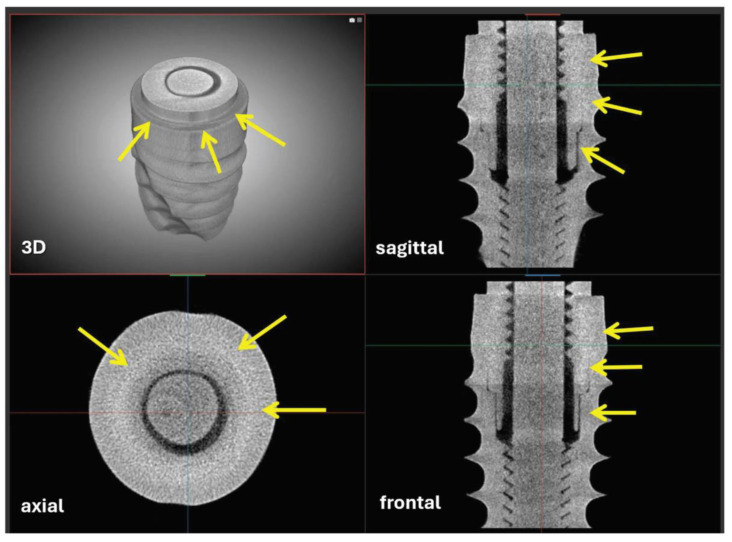
Dental titanium implant with conometric connection: 3D reconstruction and sampling sagittal, axial, and frontal slices. Freeze frame from Supplementary Video S1. The connection interface (yellow arrows) is free of macro- and micro-gaps.

The micro-CT analysis carried out on nr.2 commercial implants as such (and in static conditions) revealed the absence of macro- and micro-gaps for both samples studied; in fact, as shown in Figure 5 and Video S1 of the Supplementary Materials, the connection interface (indicated with the yellow arrows in Figure 5) cannot be viewed in any of the three axial, sagittal, and frontal perspectives. This result is extremely satisfactory in order to guarantee the in-service stability of the implant, for the benefit of the peri-implant bone, and to prevent bacterial infiltration.

### 3.2. Synchrotron Micro-CT

**Figure 6:**
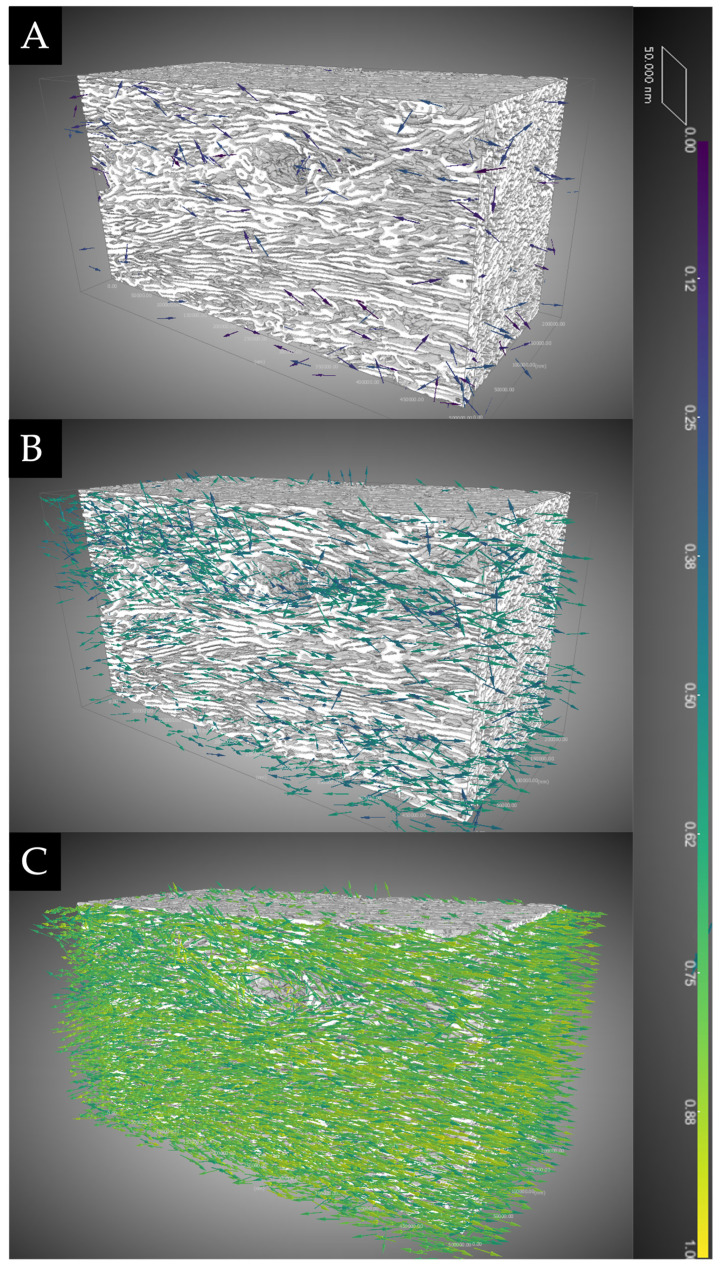
Qualitative analysis of the degree of anisotropy (DA). The depicted eigenvectors serve to differentiate between (**A**) regions characterized by low DA (0–0.30), (**B**) regions with medium DA (0.30–0.60), and (**C**) regions with high DA (0.60–1.0).

The analysis of the degree of anisotropy (DA) provided us with a comprehensive examination of the extent to which the collagen bundles were oriented. The eigenvectors depicted in Figure 6 serve to differentiate between regions characterized by low DA (0–0.30), regions with medium DA (0.30–0.60), and regions with high DA (0.60–1.0). The number of eigenvectors was notably greater within the maximal discriminant analysis range; this observation indicates that the collagen bundles had a distinct orientation and were highly aligned. As a result, with the assistance of artificial intelligence, it became feasible to discern the primary orientations and quantify them.

**Figure 7:**
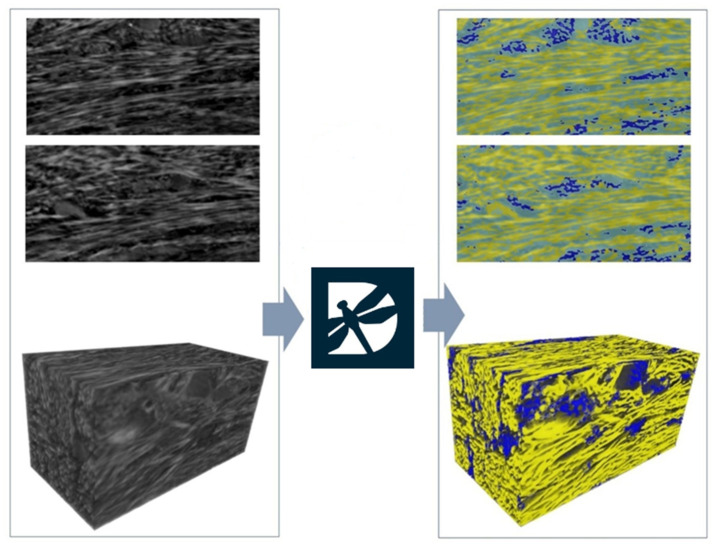
Segmentation achieved by artificial intelligence algorithms. Left panels: two raw axial slices and a 3D rendering of a subvolume as achieved after phase-retrieval 3D reconstruction. Right panels: semantic segmentation of the same slices and 3D rendering with deep learning. In semantic segmentation, the transversal bundles were rendered in yellow, the longitudinal bundles were rendered in blue, and the background was rendered in teal. Data were processed with Dragonfly software (Vers. 2022.1; Object Research Systems, Montreal, QC, Canada); semantic segmentation was performed by the U-Net neural network.

The semantic segmentation of the images obtained from the synchrotron analyses allowed us to discriminate the transverse from the longitudinal bundles, as shown in Figure 7, adding more quantitative information for these two classes that could not be distinguished with the common thresholding techniques. The quantitative analyses of the four different sub-volumes segmented by the U-Net neural network in the deep learning tool allowed us to achieve the quantification of transverse bundles that were found to be significantly higher than the longitudinal ones (45.4 ± 2.5% vs. 5.3 ± 1.6%, respectively; *p* < 0.0001). The anisotropy degree (DA) was found to be significantly lower for the longitudinal bundles than for the transversal ones (0.834 ± 0.017 vs. 0.747 ± 0.009, respectively; *p* < 0.001). Moreover, with reference to the connectivity density parameter (Conn. D), it showed significantly higher values in the transverse direction than in the longitudinal one (2.77 ± 0.31 vs. 0.12 ± 0.11 (×10^−4^) px^−3^, respectively; *p* < 0.001).

### 3.3. Histology and Polarized Light Microscopy

At low magnification, soft tissues and newly formed bone were observed around the implant abutment. The peri-implant soft tissues adhered to the abutment surface and there were no gaps at the interface. They were composed of sulcular epithelium (SE), junctional epithelium (JE), and connective tissue (CT). The JE was composed of a layer of epithelial cells in close contact with the surface of the transgingival collar.

In the apical portion, the CT was close to the surface of the stump. It was 610 µm high and characterized by the presence of some small blood vessels. At higher magnification, areas of newly formed bone, up to 2080 µm above the implant shoulder, were found. Specifically, newly formed trabecular bone grew over the implant shoulder and had a direction up to the abutment surface. A layer of connective tissue was present between the newly formed bone and the surface of the abutment; no epithelium was observed in this portion. The tissue height from the implant shoulder (IS) to the margin of peri-implant mucosa (MP) was approximately 5080 µm.

**Figure 8:**
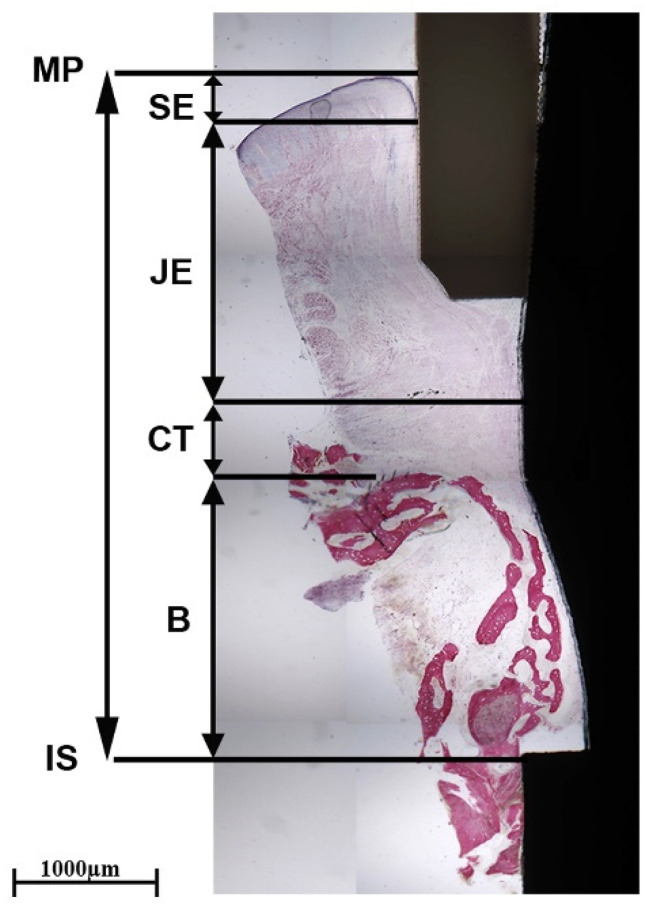
Histological section of the implant–abutment unit. Peri-implant soft tissues (MP) were composed of sulcular epithelium (SE), junctional epithelium (JE), and connective tissue (CT), which were in close contact with the abutment surface. The new bone formation (B) had grown above the implant shoulder (IS) (acid fuchsin–toluidine blue 20×).

**Figure 9:**
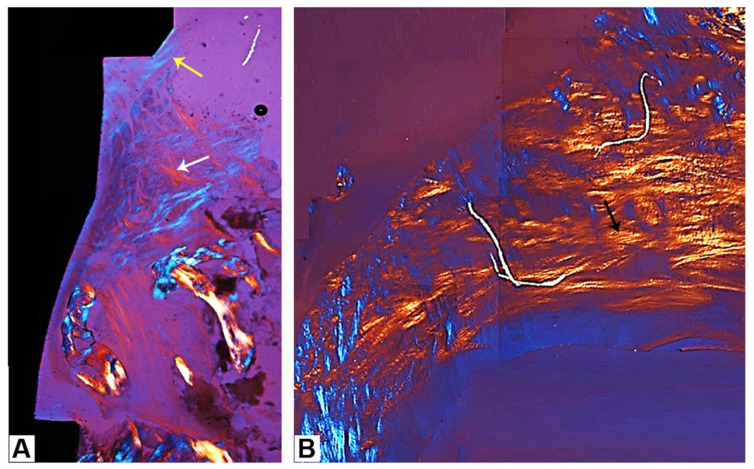
Polarized light microscopy of the peri-implant soft tissues: (**A**) longitudinal bundles distributed parallel to the surface of the abutment (yellow arrow) in the coronal portion, while in the apical portions—and far away from the implant surface—there was a lattice composed of interwoven bundles (white arrow); (**B**) the transverse section with the majority of semicircular collagen bundles (black arrow) around the abutment (acid fuchsin–toluidine blue 40×).

The peri-implant mucosa included several collagen bundles, which were visible in both longitudinal and transverse sections, as shown during polarized light microscopy. As far as the longitudinal section was concerned, in the portions close to the new bone, longitudinal bundles were observed parallel to the profile of the implant and the abutment in the long axis (Figure 8 and Figure 9A). Regarding the cross-section, predominantly semicircular fibers could be observed around the abutment (Figure 9B). 

## 4. Discussion

The causes of dental implant failure can be divided into two broad categories: biological (for instance, peri-implantitis) or mechanical (due to fracture of the implant, fracture of the abutment, loosening or loss of the connecting screw, and fracture of the prosthetic structure). The presence of an incorrect coupling, i.e., misfit between the implant and the abutment, is known to be the basis for an increase in mechanical stress on the connection structures and the surrounding bone tissue. This condition can lead to biological consequences due to bacterial penetration into the gap that occurs between the fixture and the abutment.

While many authors have reported on the importance of the characteristics of the fixture–abutment interface on the level and loss of peri-implant bone tissue [35], few studies have examined the structure and ultrastructure of human peri-implant soft tissues [7,11,13,14,15,16]. The 3D networks of collagen bundles that compose the connective portion have been found to be transversely and longitudinally oriented in relation to the dental implant axis. During the growth of these bundles, they organize themselves into intertwining patterns [13,14,15] with different quantitative distributions depending on time from surgery, environmental conditions, and implant macro- and micro-geometry; these boundary conditions are currently the focus of several studies [7,11,13,14,15,16].

A conometric prosthetic restoration system has been reported by some authors as a means to fix the prosthetic restoration to the abutment, obviating the use of retention through the use of screws or cement. The use of this technique offers very good clinical results; however, while we were able to find relevant histomorphometric evidence of the good performance of peri-implant crestal bone [29], no definitive evidence of the 3D structure of peri-implant connective tissues around conometric prosthetic restorations has been documented.

In the present case study, through the use of micro-CT, the total absence of 3D gaps in the investigated conometric abutment–prosthetic cap connection was verified. This is extremely relevant for the in vivo service of the implant because it protects the implant from bacterial contamination.

Histological analysis of the peri-implant tissues around this human-retrieved conometric connection implant showed a lack of inflammation, most likely due to a reduced percentage of bacterial leakage when using this type of implant–abutment connection. This is in agreement with previous studies [7,8]; indeed, the few studies that have reported on bacterial contamination at the abutment–prosthesis coupling level demonstrate either an absence of a complete bacterial seal or no bacterial infiltration into and from this coupling. One of these studies reported an in vitro investigation of the microleakage of bacteria in three different implant connections for a period of 14 days [36]; in this case, 60 dental implants were distinguished into three groups, according to the type of connection: external hexagon, internal hexagon, and conometric connection. All implants were immersed in a bacterial suspension into the surrounding solution. Less bacterial leakage and a lower rate of infiltration were found in conometric connections when compared to hexagon connections. Moreover, other microbiological studies confirmed a very low percentage of bacterial leakage in conometric connection Cone Morse implants [37,38]. These results could, most likely, be related to a very precise fit between the two components, as was shown through the use of micro-CT in the present study.

Furthermore, in the present investigation, with reference to the sample implanted in vivo for a period of 45 days, growth of supracrestal bone over the shoulder of the implant was observed. This was also observed in another study on dogs [39] aiming to evaluate the histological and histomorphometric differences at the marginal bone level when using two different implant–abutment assembly designs (namely the traditional external hexagon and the Morse Cone tapered connections). In this case, it was shown that subcrestal placement had a positive impact on crestal bone remodeling in Morse Cone implants.

In the sample implanted in vivo for a period of 45 days, a large amount of correctly organized peri-implant connective tissue was also observed through synchrotron investigation and subsequent analysis of 3D morphometric parameters. It was found that the quantity and connectivity of transverse bundles were greater than those of longitudinal bundles; furthermore, it was found that the transverse bundles were better aligned than the longitudinal ones. This is in line with a previous study that found that even in mature and functional peri-implant connective tissue, the proportion between longitudinal and transverse fibers is always against the former [15].

Despite the promising results obtained in this single case study, it is necessary to carry out further tests in a statistically significant number of conometric prosthetic connections, both in static conditions—to confirm the present evidence—and in cases subjected to chewing load. Indeed, as suggested in previous studies [40,41], including synchrotron-based ones [42,43], elucidation of the mode of mechanical behavior of the implant–abutment connection under various loading scenarios will provide information to enhance the design and function of the connections and minimize the in-service failure sometimes encountered to date.

## 5. Conclusions

According to the authors’ best knowledge, this clinical case constitutes the first quantitative analysis of the 3D microarchitecture and properties of the peri-implant connective tissues surrounding a conometric prosthetic connection with proven absence of macro- and micro-gaps in static conditions.

The peri-implant soft tissues, as revealed by cross-linked light microscopy and synchrotron-based imaging, appeared to be rich in collagen bundles, with a functional intertwined organization, and tightly adhered to the abutment surface. Thus, this intertwined organization of the collagen bundles, already after just 45 days from implant surgery, seems promising and worthy of further study with the objective of searching for a favorable stabilization of the soft tissues, which could thus constitute an effective barrier against the apical migration of the inflammatory cells towards the bone.

## Data Availability

The data presented in this study are available from the corresponding author upon request due to the large size of the imaging raw data and datasets.

## Supplementary Materials

The following supporting information can be downloaded at: https://www.mdpi.com/article/10.3390/clinpract14020043/s1, Table S1: CARE Checklist of information to include when writing a case report. Video S1: Dental titanium implant with conometric connection: 3D reconstruction and sequence of sagittal, axial, and frontal slices.
