# Supplementary material for: Case Report of a Dental Implant with Conometric Abutment–Prosthetic Cap Connection: Advanced High-Resolution Imaging and Peri-Implant Connective Tissue Performance
Source: Clin Pract. 2024 Mar 27;14(2):556–69. doi: 10.3390/clinpract14020043 (PMC11049280; doi:10.3390/clinpract14020043)
Supplement: Supplementary file 1 [file clinpract-14-00043-s001.zip › clinpract-2912699-supplementary.pdf]

CARE case report guidelines

CARE Checklist of information to include when writing a case report

(cc) BY-NC-ND

Topic	Item	Checklist item description	Reported on Line
Title	1	The diagnosis or intervention of primary focus followed by the words "case report"	2-4
Key Words	2	2 to 5 key words that identify diagnoses or interventions in this case report, including "case report"	41-42
Abstract (no references)	3a	Introduction: What is unique about this case and what does it add to the scientific literature?	22-27
	3b	Main symptoms and/or important clinical findings	N.A.
	3c	The main diagnoses, therapeutic interventions, and outcomes	32-37
	3d	Conclusion—What is the main "take-away" lesson(s) from this case?	38-40
Introduction	4	One or two paragraphs summarizing why this case is unique (**may include references**)	75-82
Patient Information	5a	De-identified patient specific information.	87-130
	5b	Primary concerns and symptoms of the patient.	N.A.
	5c	Medical, family, and psycho-social history including relevant genetic information	N.A.
	5d	Relevant past interventions with outcomes	N.A.
Clinical Findings	6	Describe significant physical examination (PE) and important clinical findings.	87-130
Timeline	7	Historical and current information from this episode of care organized as a timeline	N.A.
Diagnostic Assessment	8a	Diagnostic testing (such as PE, laboratory testing, imaging, surveys).	172-230
	8b	Diagnostic challenges (such as access to testing, financial, or cultural)	N.A.
	8c	Diagnosis (including other diagnoses considered)	106-113
	8d	Prognosis (such as staging in oncology) where applicable	N.A.
Therapeutic Intervention	9a	Types of therapeutic intervention (such as pharmacologic, surgical, preventive, self-care)	87-130
	9b	Administration of therapeutic intervention (such as dosage, strength, duration)	103-106
	9c	Changes in therapeutic intervention (with rationale)	106-113
Follow-up and Outcomes	10a	Clinician and patient-assessed outcomes (if available)	N.A.
	10b	Important follow-up diagnostic and other test results	157-301
	10c	Intervention adherence and tolerability (How was this assessed?)	N.A.
	10d	Adverse and unanticipated events	N.A.
Discussion	11a	A scientific discussion of the strengths AND limitations associated with this case report	302-378
	11b	Discussion of the relevant medical literature **with references**.	302-378
	11c	The scientific rationale for any conclusions (including assessment of possible causes)	380-391
	11d	The primary "take-away" lessons of this case report (without references) in a one paragraph conclusion	380-391
Patient Perspective	12	The patient should share their perspective in one to two paragraphs on the treatment(s) they received	N.A.
Informed Consent	13	Did the patient give informed consent? Please provide if requested	Yes ☒ No ☐
